# Fabrication of Stacked MoS_2_ Bilayer with Weak Interlayer Coupling by Reduced Graphene Oxide Spacer

**DOI:** 10.1038/s41598-019-42446-w

**Published:** 2019-04-11

**Authors:** Hye Min Oh, Hyojung Kim, Hyun Kim, Mun Seok Jeong

**Affiliations:** 1 Department of Energy Science, Sungkyunkwan University (SKKU), Suwon, 16419 Republic of Korea; 2 Center for Integrated Nanostructure Physics, Institute for Basic Science (IBS), Suwon, 16419 Republic of Korea

## Abstract

We fabricated the stacked bilayer molybdenum disulfide (MoS_2_) by using reduced graphene oxide (rGO) as a spacer for increasing the optoelectronic properties of MoS_2_. The rGO can decrease the interlayer coupling between the stacked bilayer MoS_2_ and retain the direct band gap property of MoS_2_. We observed a twofold enhancement of the photoluminescence intensity of the stacked MoS_2_ bilayer. In the Raman scattering, we observed that the E^1^_2g_ and A_1g_ modes of the stacked bilayer MoS_2_ with rGO were further shifted compared to monolayer MoS_2_, which is due to the van der Waals (vdW) interaction and the strain effect between the MoS_2_ and rGO layers. The findings of this study will expand the applicability of monolayer MoS_2_ for high-performance optoelectronic devices by enhancing the optical properties using a vdW spacer.

## Introduction

The recent discovery of a new class of two-dimensional (2D) materials, transition metal dichalcogenides (TMDs), such as molybdenum disulfide (MoS_2_) and tungsten disulfide (WS_2_), has attracted attention because of their unique layer-dependent electrical and optical properties^1–3^. For example, MoS_2_ possesses an indirect band gap of ~1.29 eV in bulk, but it becomes a direct optical band gap of ~1.90 eV in the monolayer and can affect the electronic and optical properties^4,5^. These interesting features in MoS_2_ have opened up new possibilities for optoelectronic applications^6^. Previous reports have mainly focused on the fabrication of optoelectronic devices, such as phototransistors, photodetectors, light-emitting diodes, and solar cells with monolayer MoS_2_^7–10^. However, the absorbance of monolayer MoS_2_ is not strong enough to realize efficient optoelectronic devices^4^. Compared to the monolayer, bilayer or few-layer MoS_2_ shows improved absorbance and carrier mobility^11^. However, few-layer MoS_2_ has an indirect band gap that limits its applicability for high-efficiency optoelectronic devices. Therefore, many researchers have worked to improve the mobility and intensity of the photoluminescence (PL) of MoS_2_ with doping, strain, and defect engineering^12–14^. More recently, it was reported that artificially stacked TMD with a spacer between the individual layers can improve the optical properties while maintaining the intrinsic properties of TMD^15–18^. Among them, Piljae Joo *et al*. demonstrated an enhancement of the photoluminescence (PL) of stacked few-layer MoS_2_ with a polymer spacing layer. However, this method has been realized with few-layer or multilayer MoS_2_^15^. For the stacking of monolayer TMD, hexagonal boron nitride (h-BN) was introduced as the spacer for the TMD hetero-bilayer or homo-bilayer^16–18^. These results suggest that stacked TMD with the h-BN layer can retain the direct band gap feature of the monolayer TMD. However, the procedure for transferring h-BN onto TMDs is somewhat complicated; thus, it is difficult to apply it to large areas. Therefore, it is essential to devise a simple technique that can be implemented easily into the device-fabrication process to obtain higher optoelectronic properties.

Here, we demonstrate the fabrication of stacked MoS_2_ with a reduced graphene oxide (rGO) spacer. rGO has remarkable properties, such as high thermal conductivity, electrical conductivity, and mechanical stability^19^. rGO can decrease the interlayer coupling of stacked MoS_2_, such that rGO is an ideal sheet to decouple the top and bottom MoS_2_ and retain the direct band gap property of MoS_2_. We observed an enhancement of the PL intensity of stacked MoS_2_ with an rGO spacer compared to that of monolayer MoS_2_. We also systematically investigated the origin the structural, chemical, and optical properties of the stacked MoS_2_ with/without spacer layer.

## Results and Discussion

**Figure 1:**
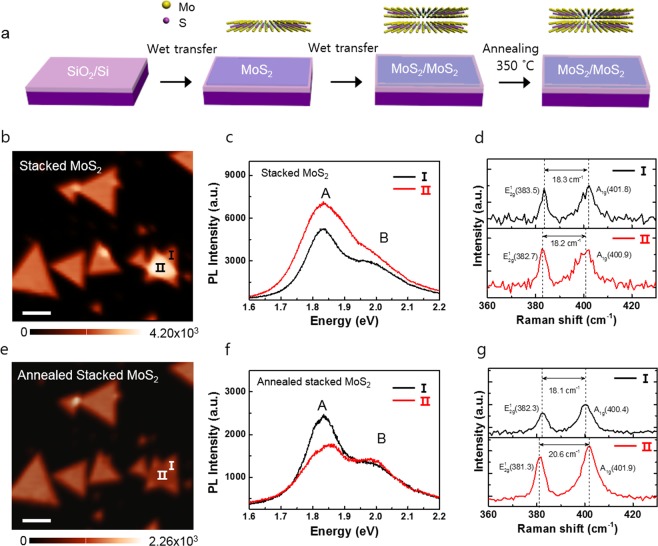
(**a**) schematic of sample preparation of stacked MoS_2_ with/without thermal annealing. PL intensity map and PL and Raman spectra of I and II region in the stacked MoS_2_ (**b**–**d**) before and (**e**–**g**) after annealing.

Figure 1a illustrates the preparation process of stacked MoS_2_. The chemical vapor deposition (CVD)-grown monolayer MoS_2_ films were transferred onto the 300-nm-thick of SiO_2_/Si substrate using the conventional wet transfer method. (See Methods section for details) To prepare stacked bilayer MoS_2_, another CVD-grown monolayer MoS_2_ film was transferred onto the monolayer-MoS_2_/SiO_2_/Si template. First, we confirmed the basic optical properties of the monolayer MoS_2_ by using the PL and Raman analysis (Supporting Information (SI), Fig. S1). Figure 1b shows the PL intensity map of monolayer MoS_2_ flakes and stacked MoS_2_ flakes. Normally, the PL intensity of bilayer MoS_2_ is much lower than that of monolayer MoS_2_ because it has an indirect band gap^4,5^.

Interestingly, we observed that the integrated PL intensity of stacked MoS_2_ flake (II) is slightly higher than that of the monolayer MoS_2_ flake (I). It is implied that stacked bilayer MoS_2_ flake has different optical features that are irrelevant to the layer-dependent optical properties of MoS_2_.

To confirm the PL peak position of the sample, we extracted the PL spectrum from the stacked MoS_2_ flakes marked I and II in Fig. 1b. The A and B exciton peak positions at ~1.83 and ~1.98 eV of I and II were almost the same (Fig. 1c). Figure 1d shows the Raman spectra of I and II. We observed the two Raman modes at approximately 383.5 and 401.8 cm^−1^ of monolayer region I in the stacked MoS_2_, corresponding to the E^1^_2g_ and A_1g_ modes^20^. The distance between the E^1^_2g_ and A_1g_ modes was 18.3 cm^−1^. The A_1g_ and E^1^_2g_ modes of the region II were shifted compared to those of region I, but the distance between the two Raman modes is around 18.2 cm^−1^, which is related to the monolayer properties of MoS_2_^20^. We confirmed the same trend from other stacked MoS_2_ flakes, regardless of stacking angle (see SI, Fig. S2). Figure 1e shows the PL intensity map of the same stacked MoS_2_ sample after thermal annealing at ~300 °C for 1 h under nitrogen gas. After thermal annealing, the integrated PL intensity was reduced for all of the stacked MoS_2_ flakes. The PL intensity of region II is decreased compared to that of region I. In region II of stacked MoS_2_, the intensity of exciton peak A decreases significantly and is slightly shifted (Fig. 1f). These PL results show a similar characteristic to the natural bilayer^21,22^. In addition, the distance between the E^1^_2g_ and A_1g_ modes increased from ~18.2 to ~20.6 cm^−1^, which is somewhat similar to the bilayer properties (Fig. 1g)^20^. From the PL and Raman results, we found that annealed stacked MoS_2_ regions have bilayer properties.

**Figure 2:**
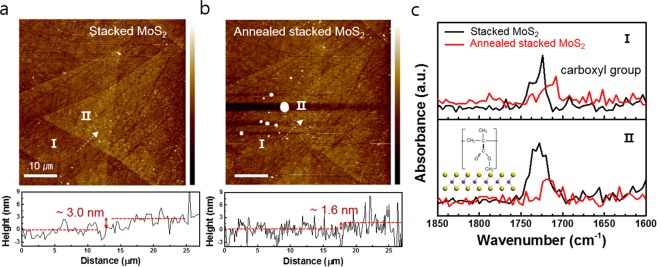
(**a**,**b**) is AFM height image of the stacked bilayer MoS_2_ before and after annealing. (**c**) Absorbance spectra of each position of stacked bilayer MoS_2_ before and after annealing treatment.

**Figure 3:**
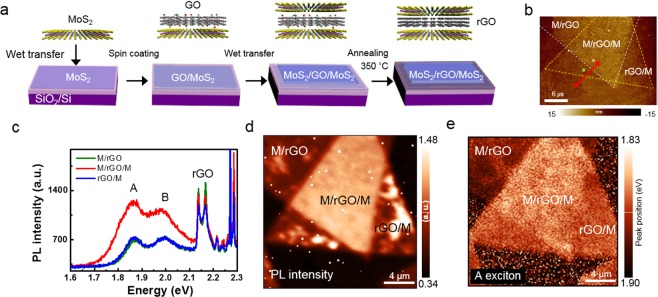
(**a**) Illustration for sample preparation of stacked MoS_2_ with rGO. (**b**) AFM topography image of the stacked MoS_2_ with rGO. (**c**) PL spectra of the various position of the stacked MoS_2_ with rGO. (**d**) PL intensity map image of stacked MoS_2_ with rGO for 1.67~2.11 eV corresponding to the MoS_2_. (**e**) PL peak position map image of the stacked MoS_2_ with rGO.

To investigate the phenomena of the different optical properties of the stacked MoS_2_ before and after thermal annealing, we carried out photothermal induced infrared resonance (PTIR) spectroscopy, which simultaneously provides topographical information and infrared absorbance (see SI, Fig. S3)^23^. Fig. 2a,b show the AFM topography images and height profiles of stacked MoS_2_ before and after thermal annealing, respectively. The topography images provide distinguishable contrast between the monolayer (I) and stacked (II) MoS_2_ region. We measured the thickness of the stacked MoS_2_ from region I to II. The thickness of the dashed line at the stacked MoS_2_ before thermal annealing was ~3.0 nm. This value is much larger than the expected thickness of the stacked bilayer MoS_2_^22^. However, after thermal annealing, the thickness of the stacked MoS_2_ decreased to ~1.6 nm, which is almost identical to the thickness of the bilayer MoS_2_^22^. According to the previous results, it is assumed that defects or organic molecules exist in between the stacked flakes^21^. Fig. 2c presents the PTIR absorbance spectra of regions I and II of the stacked MoS_2_ sample before and after thermal annealing treatment. We observed a peak at approximately 1728 cm^−1^, corresponding to the C=O mode of the poly(methyl methacrylate) (PMMA) residues on the overall area of the stacked MoS_2_ sample before thermal annealing^24^. Normally, PMMA is used as a supporting material during the wet-transfer process. However, polymer residues usually remain on the surface of the TMD thin film after the transfer process, and it is difficult to completely remove them from the TMD thin film^21,25^. Therefore, the stacked bilayer MoS_2_ are thicker than the original bilayer MoS_2_ because polymer residues are present on MoS_2_. The polymer residue suppresses the interlayer interaction between stacked MoS_2_ monolayers. Thus, the PL and Raman results of stacked MoS_2_ before annealing showed monolayer characteristics (Fig. 1b–d). In contrast, after thermal annealing, the intensity of the C=O mode decreased. This indicates that the concentration of the PMMA is reduced (Fig. 2c). The thermal annealing removed the polymer residues and caused interlayer interaction in stacked MoS_2_. Thus, the thickness of stacked MoS_2_ decreased after annealing, and it exhibited bilayer characteristics. From these results, the polymer residues between stacked MoS_2_ flakes can act as a spacer, which can retain the intrinsic properties of monolayer MoS_2_ prior to thermal annealing. However, during the device-fabrication process, the thermal annealing process inevitably decreases the resistivity of the interface for better device performance^26^. Therefore, it is necessary to find the optimum spacer in the stacked MoS_2_ that blocks the intercoupling between flakes without damage in the thermal annealing process. Figure 3a shows an illustration of the sample-preparation process of the stacked MoS_2_ with spacer layer. The CVD-grown monolayer MoS_2_ films were transferred onto the 300-nm SiO_2_/Si substrate using the wet-transfer method. The prepared GO solution was spin-coated at 500 rpm for 5 s, followed by 1500 rpm for 60 s on top of the monolayer MoS_2_ on the SiO_2_/Si substrate. The average thickness of the coated rGO on the MoS_2_ sheet was approximately 5 ± 2 nm (see SI, Fig. S4). To prepare hybrid stacked MoS_2_, another CVD-grown monolayer MoS_2_ film was transferred onto the GO-coated monolayer MoS_2_/SiO_2_/Si template. By thermal annealing treatment at 350 °C for 3 h, we fabricated the stacked MoS_2_ with an rGO spacer. To investigate the reduction of GO, we carried out Raman spectroscopy measurement of the GO before and after thermal annealing (see SI, Fig. S5). Figure 3b is the AFM topography image of the stacked MoS_2_ with rGO spacer. According to the AFM topography image, the monolayer and stacked MoS_2_ region can be distinguished. Additionally, we observed wrinkles and some bubbles on the stacked MoS_2_ with rGO sample. These seems to be formed during the transfer process (see SI, Fig. S6). The thickness of the monolayer MoS_2_ with rGO (green dot) is approximately 1.1 nm and that of the stacked bilayer MoS_2_ with rGO region (orange dot) is approximately 2.7 nm (see SI, Fig. S7). For the spatially resolved optical characterization, confocal PL and Raman measurements were performed for stacked MoS_2_ with rGO spacer. Figure 3c shows the PL spectra obtained from each position of the sample. We observed the A and B excitons (~1.86 and 2.0 eV) of MoS_2_ and the Raman G and D bands (~2.13 and 2.16 eV) of the rGO at PL spectra^12^. As shown in Fig. 3c, the PL intensity of stacked bilayer MoS_2_ with rGO (M/rGO/M) region is twice that of rGO on monolayer MoS_2_ (rGO/M) or monolayer MoS_2_ on rGO (M/rGO) regions. In previous reports, PL quenching was observed in MoS_2_ over mechanically exfoliated graphene (MEG) because MEG has semi-metallic properties that result in a metal-semiconductor interface^27,28^. On the other hand, rGO show semiconductor behavior because of residual oxygen functional groups after thermal annealing^29,30^. Therefore, PL quenching is not observed in the interface between rGO and MoS_2_ stacked structure. Figure 3d represents the PL intensity map for the energy range of 1.67–2.11 eV of stacked bilayer MoS_2_ with rGO spacer. The PL intensity map could also be used to distinguish the stacked M/rGO/M, M/rGO, and rGO/M regions because these PL intensity values are different from each other. As observed in Fig. 3d, some position of rGO/M region has a higher PL intensity rather than the M/rGO/M region. To compare the intensity, we extracted the PL spectra of bright spot of rGO/M region and M/rGO/M region (see SI, Fig. S8). According to previous report, GO induce the p-type doping of monolayer MoS_2_ because of the functional groups of GO^12^. We believe that the cause of high PL intensity in the rGO/M region is p-type doping by residue of functional group after thermal annealing. A slight red shift in the A exciton peak is observed in the M/rGO/M region. In addition, the PL peak position of the stacked MoS_2_ with rGO sample is different (Fig. 3e). We confirmed that the PL results at stacked MoS_2_ with rGO spacer showed different characteristics from those of the stacked MoS_2_ without rGO spacer.

**Figure 4:**
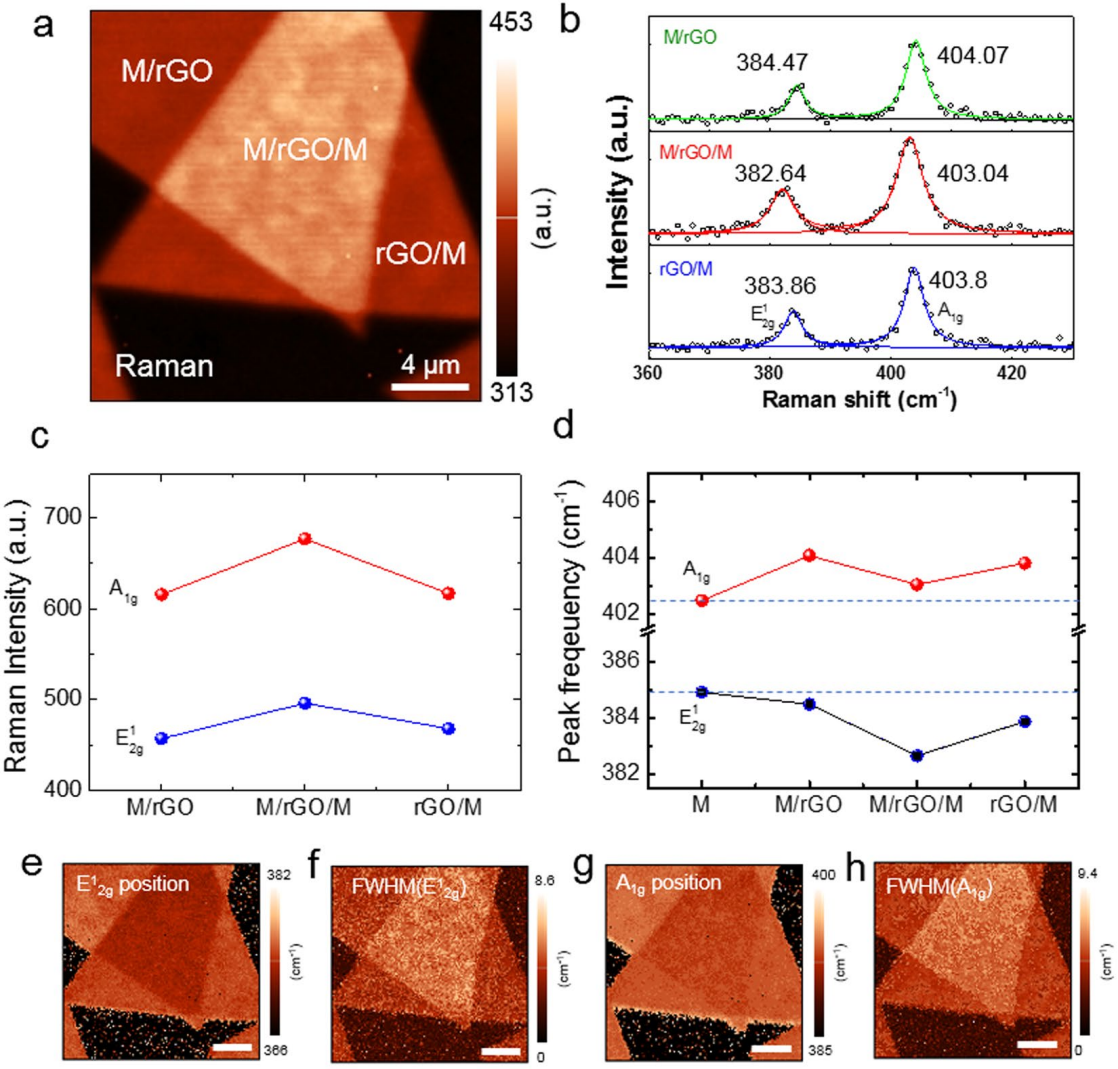
(a) Raman intensity map of the stacked MoS_2_ with rGO. (**b**) Local Raman spectra of various position of the stacked MoS_2_ with rGO. (**c**) Raman intensity and (**d**) peak position of the E^1^_2g_ and A_1g_ of the various position of stacked MoS_2_ with rGO. (**e**) Peak position and FWHM map of E^1^_2g_ (**f**) peak position and FWHM map of the A_1g_ of the sample.

To clarify the cause for the considerably changed optical and structural properties of stacked MoS_2_ with rGO spacer, we have performed confocal Raman mapping. Figure 4a shows the Raman intensity map of the stacked MoS_2_ with rGO. The Raman intensity map provides distinguishable contrast between the stacked and monolayer MoS_2_ regions. Figure 4b shows the local Raman spectra of each position. The peak positions of the two Raman modes of stacked MoS_2_ with rGO spacer are different from those of the stacked MoS_2_ without spacer. The A_1g_ and E^1^_2g_ modes of stacked MoS_2_ with rGO were further shifted compared to those of stacked MoS_2_ without spacer. In order to understand these results, we focus on the E^1^_2g_ and A_1g_ modes of MoS_2_, depending on the region. Figure 4c presents the Raman intensity of the E^1^_2g_ and A_1g_ modes of the MoS_2_ according to each position. The higher Raman intensity of the M/rGO/M region originates from the increased scattering cross section by stacked MoS_2_ with rGO. Figure 4d shows the peak position of the two Raman modes according to the position of the stacked MoS_2_ with rGO spacer. The E^1^_2g_ and A_1g_ modes of the stacked MoS_2_ with rGO spacer were observed to shift to the opposite direction compared to that of pristine monolayer MoS_2_ (M). Interestingly, we observed that the red shift of E^1^_2g_ on M/rGO/M is large compared to M/rGO and rGO/M, which is shown in the Raman peak position map in Fig. 4e. Furthermore, the full width at half maximum (FWHM) of the E^1^_2g_ peak of M/rGO/M broader than that of other regions (Fig. 4f). This indicates that interfacial strain is generated more on the M/rGO/M than on the M/rGO and rGO/M^31,32^. In contrast, the blue shift of A_1g_ on M/rGO and rGO/M is larger than the shift observed in M/rGO/M, which is confirmed in the Raman map shown in Fig. 4g. The FWHM of the A_1g_ peak of M/rGO/M slightly broader than that of other regions (Fig. 4h). This means that the van der Waals (vdW) interlayer interaction between the MoS_2_ and rGO on M/rGO and rGO/M is stronger than that of M/rGO/M^31^. According to the previous results, the blue shift of A_1g_ of MoS_2_ can originate from the p-doping effect by the functional group of GO^12,33^. The A_1g_ peak appeared to be more strongly shifted as the amount of GO functional groups increased^12^. In this study, rGO was prepared with GO by thermal annealing. Thus, most functional groups of GO were removed and only a small amount of the functional group remained on the surface^25^. In addition, we confirmed that the A_1g_ mode of stacked MoS_2_ with rGO was further shifted compared to stacked MoS_2_ with GO (see SI, Fig. S9). This means that p-doping of MoS_2_ by rGO has a negligible effect on the shift of the A_1g_ peak observed in our stacked MoS_2_ with rGO. Therefore, it is concluded that the stacked MoS_2_ with rGO spacer is in close contact with vdW interaction between MoS_2_ and rGO. We suggest that the rGO can serve as a spacer to decouple the top and bottom MoS_2_ and retain the direct band gap of MoS_2_.

## Conclusions

In conclusion, we successfully fabricated stacked bilayer MoS_2_ with rGO spacer. We observed that the decreased PL intensity in the stacked MoS_2_ layers without spacer because of bilayer properties. Meanwhile, when the rGO is used as a spacer in between MoS_2_ sheets, PL intensity shows sum up of PL from two individual MoS_2_ sheets. It clearly indicates that rGO is a suitable material to decouple the top and bottom MoS_2_ and retain the direct band gap of MoS_2_. In the Raman results, we observed the vdW interaction and the strain effect between the MoS_2_ and rGO layers. We expect that these results will provide a fundamental understanding of the interlayer interaction of stacked 2D materials and enable further development of stacked MoS_2_-based devices.

## Methods

### Conventional wet transfer method

Poly(methyl methacrylate) (PMMA) (Micro Chem, 4 wt% in chlorobenzene) was coated onto monolayer MoS_2_ grown on a SiO_2_/Si substrate to serve as a supporting layer for the transfer process. After being coated with PMMA, the sample was floated on a 1 M potassium hydroxide (KOH) solution at 80 °C to remove the SiO_2_ layer. Subsequently, only monolayer MoS_2_ with PMMA remained on the KOH solution. The remaining PMMA/MoS_2_ was washed with DI water to remove any residual KOH etchant. The washed PMMA/MoS_2_ was transferred to the SiO_2_/Si substrate. After the drying process was complete, the PMMA was removed using acetone.

### Preparation of the GO

GO was synthesized from natural graphite (Alfa Aesar, 99.999% purity, 200 mesh) by modified Hummers’ method. First, 5 g of graphite powder and 350 mL of 10 M Sulfuric acid (H_2_SO_4_) were blended. KMnO_4_ (15 g) was slowly added over approximately an 1 h. Stirring was continued for 2 h in a cooled water bath. The mixture was strongly stirred for 3 days at room temperature. Deionized water was added and stirring for 10 min. The mixture was stirred for 2 h at room temperature after the addition of an aqueous solution of H_2_O_2_ (30 wt%). Aqueous solution of HCl (35 wt%) was then added and stirred for 30 min at room temperature. After the supernatant solution was decanted, deionized water was slowly added and stirred for 30 min. The GO solution 1 g/l in water was sonicated for 1 h to exfoliate the GO sheets. To obtain dispersed GO, centrifugation at 10,000 rpm was performed for 1 h, and the supernatant solution was decanted.

### Characterization Methods

PL spectra were obtained using a confocal PL spectrometer equipped with an objective lens with high numerical aperture of 0.7 and a diode-pumped solid-state laser (532 nm). Confocal Raman spectroscopy was conducted using a commercial multifunctional microscope (NTEGRA, NT-MDT). The atomic force microscopy (AFM) topography image and photothermal induced infrared resonance (PTIR) absorption of the sample was obtained using a commercial Nano-IR system (Anasys Instruments).

## Supplementary information

supporting information

## References

[1] Wang QH, Kalantar-Zadeh K, Kis A, Coleman JN, Strano MS (2012). Electronics and optoelectronics of two-dimensional transition metal dichalcogenides. Nat. Nanotechnol..

[2] Zhao W (2013). Evolution of Electronic Structure in Atomically Thin Sheets of WS_2_ and WSe_2_. ACS Nano.

[3] Nayak PK, Yeh C-H, Chen Y-C, Chiu P-W (2014). Layer-Dependent Optical Conductivity in Atomic Thin WS_2_ by Reflection Contrast Spectroscopy. ACS Appl. Mater. Interfaces.

[4] Mak KF, Lee C, Hone J, Shan J, Heinz TF (2010). Atomically Thin MoS_2_ A New Direct-Gap Semiconductor. Phys.Rev.Lett..

[5] Splendiani A (2010). Emerging Photoluminescence in Monolayer MoS_2_. Nano Letters.

[6] Baugher BWH, Churchill HOH, Yang Y, Jarillo-Herrero P (2014). Optoelectronic devices based on electrically tunable p–n diodes in a monolayer dichalcogenide. Nat. Nanotechnol..

[7] Yin Z (2012). Single-Layer MoS_2_ Phototransistors. ACS Nano.

[8] Lopez-Sanchez O, Lembke D, Kayci M, Radenovic A, Kis A (2013). Ultrasensitive photodetectors based on monolayer MoS_2_. Nat. Nanotechnol..

[9] Yin Z (2014). Preparation of MoS_2_–MoO_3_ Hybrid Nanomaterials for Light-Emitting Diodes. Angew. Chem. Int. Ed..

[10] Tsai M-L (2014). Monolayer MoS_2_ Heterojunction Solar Cells. ACS Nano.

[11] Ming-Wei L (2016). Thickness-dependent charge transport in few-layer MoS_2_ field-effect transistors. Nanotechnology.

[12] Oh HM (2016). Modulating Electronic Properties of Monolayer MoS_2_ via Electron-Withdrawing Functional Groups of Graphene Oxide. ACS Nano.

[13] Castellanos-Gomez A (2013). Local Strain Engineering in Atomically Thin MoS_2_. Nano Lett..

[14] Nan H (2014). Strong Photoluminescence Enhancement of MoS_2_ through Defect Engineering and Oxygen Bonding. ACS Nano.

[15] Joo P (2014). Functional Polyelectrolyte Nanospaced MoS_2_ Multilayers for Enhanced Photoluminescence. Nano Lett..

[16] Latini S, Winther KT, Olsen T, Thygesen KS (2017). Interlayer Excitons and Band Alignment in MoS_2_/hBN/WSe_2_ van der Waals Heterostructures. Nano Lett..

[17] Yifeng C, Su Ying Q (2018). Tunable bright interlayer excitons in few-layer black phosphorus based van der Waals heterostructures. 2D Mater..

[18] Srivastava A, Fahad MS (2016). Vertical MoS_2_/hBN/MoS_2_ interlayer tunneling field effect transistor. Solid State Electron.

[19] Gao J, Liu C, Miao L, Wang X, Chen Y (2016). Free-Standing Reduced Graphene Oxide Paper with High Electrical Conductivity. J. Electron. Mater..

[20] Li H (2012). From Bulk to Monolayer MoS2: Evolution of Raman Scattering. Adv. Funct. Mater..

[21] Huang S (2014). Probing the Interlayer Coupling of Twisted Bilayer MoS_2_ Using Photoluminescence Spectroscopy. Nano Lett..

[22] Eda G (2011). Photoluminescence from Chemically Exfoliated MoS_2_. Nano Lett..

[23] Katzenmeyer AM, Aksyuk V, Centrone A (2013). Nanoscale Infrared Spectroscopy: Improving the Spectral Range of the Photothermal Induced Resonance Technique. Anal. Chem..

[24] Filimon M (2010). Smart polymer surfaces: mapping chemical landscapes on the nanometre scale. Soft Matter.

[25] Choi W, Shehzad MA, Park S, Seo Y (2017). Influence of removing PMMA residues on surface of CVD graphene using a contact-mode atomic force microscope. RSC Adv..

[26] English CD, Shine G, Dorgan VE, Saraswat KC, Pop E (2016). Improved Contacts to MoS_2_ Transistors by Ultra-High Vacuum Metal Deposition. Nano Lett..

[27] Yuan L (2016). A reliable way of mechanical exfoliation of large scale two dimensional materials with high quality. AIP Adv..

[28] Tan H (2016). Doping Graphene Transistors Using Vertical Stacked Monolayer WS_2_ Heterostructures Grown by Chemical Vapor Deposition. ACS Appl. Mater. Interfaces.

[29] Tu NDK, Choi J, Park CR, Kim H (2015). Remarkable Conversion Between n- and p-Type Reduced Graphene Oxide on Varying the Thermal Annealing Temperature. Chem. Mater..

[30] Phan D-T, Chung G-S (2013). P–n junction characteristics of graphene oxide and reduced graphene oxide on n-type Si(111). J. Phys. Chem. Solids.

[31] Zhou K-G (2014). Raman Modes of MoS_2_ Used as Fingerprint of van der Waals Interactions in 2-D Crystal-Based Heterostructures. ACS Nano.

[32] Wang Y, Cong C, Qiu C, Yu T (2013). Raman Spectroscopy Study of Lattice Vibration and Crystallographic Orientation of Monolayer MoS_2_ under Uniaxial Strain. Small.

[33] Buscema M, Steele GA, van der Zant HSJ, Castellanos-Gomez A (2014). The effect of the substrate on the Raman and photoluminescence emission of single-layer MoS_2_. Nano Res..

