# Supplementary material for: Fabrication of Stacked MoS2 Bilayer with Weak Interlayer Coupling by Reduced Graphene Oxide Spacer
Source: Sci Rep. 2019 Apr 11;9:5900. doi: 10.1038/s41598-019-42446-w (PMC6459906; doi:10.1038/s41598-019-42446-w)
Supplement: Supplementary file 1 — supporting information [file 41598_2019_42446_MOESM1_ESM.docx]

**Supporting information**

**Fabrication of Stacked MoS_2_ Bilayer with Weak Interlayer Coupling by Reduced Graphene Oxide Spacer**

Hye Min Oh^1^, Hyojung Kim^1,2^, Hyun Kim^1,2^, and Mun Seok Jeong *^1,2^

^1^Department of Energy Science, Sungkyunkwan University (SKKU), Suwon 16419, Republic of Korea

^2^Center for Integrated Nanostructure Physics, Institute for Basic Science (IBS), Suwon 16419, Republic of Korea

**Supporting Information**

S1: PL and Raman spectra of the monolayer MoS_2_

S2: PL intensity map and PL and Raman spectra stacked MoS_2_

S3: Photothermal induced infrared resonance (PTIR) spectroscopy

S4: Patterned rGO on MoS_2_ fabrication and height profile of rGO

S5: Raman spectrum of the GO before and after thermal annealing.

S6: Height profile of the stacked MoS_2_ with GO or rGO

S7: Height profile of the stacked MoS_2_ with rGO

S8: PL intensity map and PL spectra stacked MoS_2_ with rGO

S9: Raman spectra of the stacked MoS_2_ with GO or rGO.

Figure S1: PL and Raman spectra of the monolayer MoS_2_.


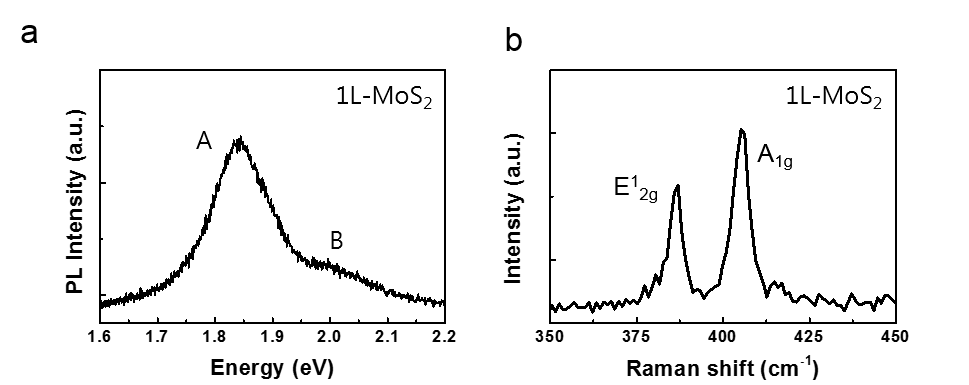


**Figure S1.** (a) PL and (b) Raman spectrum of 1L-MoS_2_

Figure S2: PL intensity map and PL and Raman spectra stacked MoS_2_.


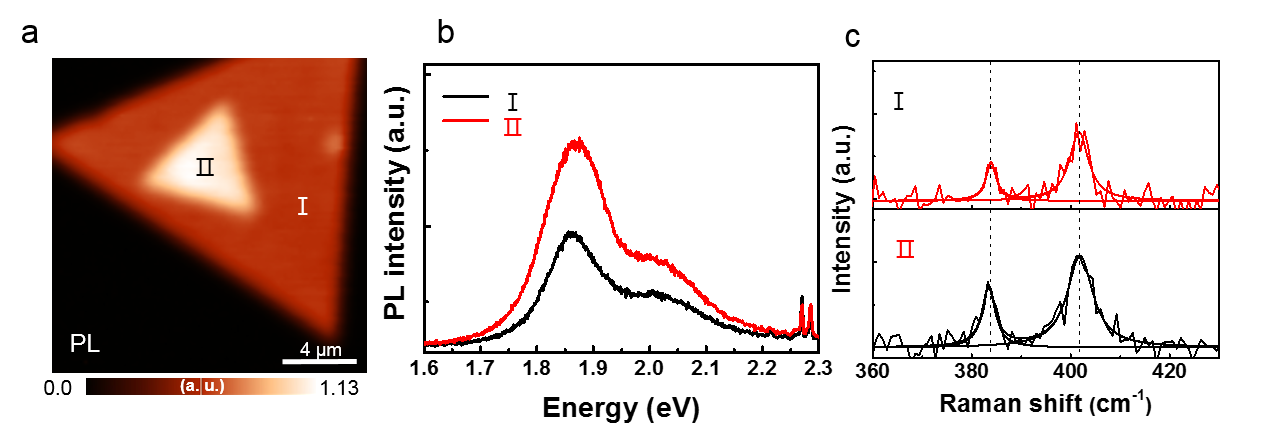


**Figure S2** (a) PL intensity map image of the stacked MoS_2_. (b) local PL spectra (c) Raman spectra of the I and II regions of the stacked MoS_2_.

Figure S3: photothermal induced infrared resonance (PTIR) spectroscopy


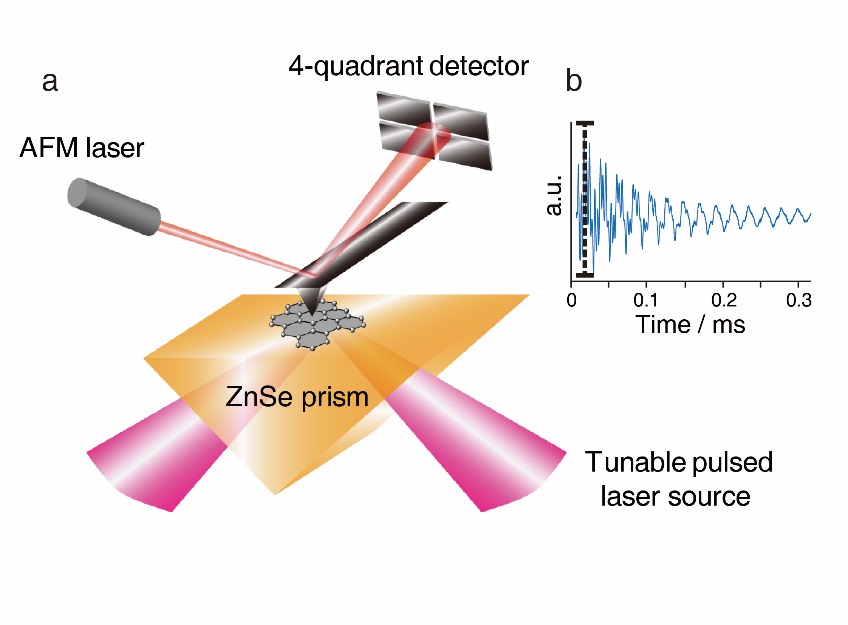


**Figure S3.** a) Schematic of the PTIR measure. (b) PTIR deflection signal of residual PMMA as a function time.

PTIR consists of an AFM which support contact mode and nanosecond tunable optical parametric oscillator (OPO) laser source with 1 kHz repetition rate tunable between 4000 cm^-1^ and ≈ 900 cm^-1^. For the nanosecond OPO laser, the pulse duration (9 ns) was kept smaller than the heat diffusion time in the sample. The sample is placed on ZnSe prism and illuminated with total internal reflection geometry while focusing the laser light with focus diameter of ~ 30 μm under the sample. The sample suffers local thermal expansion with absorbing this infrared (IR) light. Due to this expansion, AFM probe in contact mode positioned in the center of incident laser focus spot starts to deflect and oscillate with its natural frequency. This oscillation was recorded by measuring the distortion of beam path of AFM laser by four quadrant photo-detector. By this way, the local absorption of the sample can be recorded with ~100 nm spatial resolution. The absorption spectrum at this position was obtained by recording this amplitude while varying the incident laser frequency from 2000 cm^-1^ to 1000 cm^-1^. IR absorption image at certain frequency can be obtained by scanning AFM probe in contact mode while fixing incident laser frequency.^1^

Figure S4: Patterned rGO on MoS_2_ fabrication and height profile of rGO


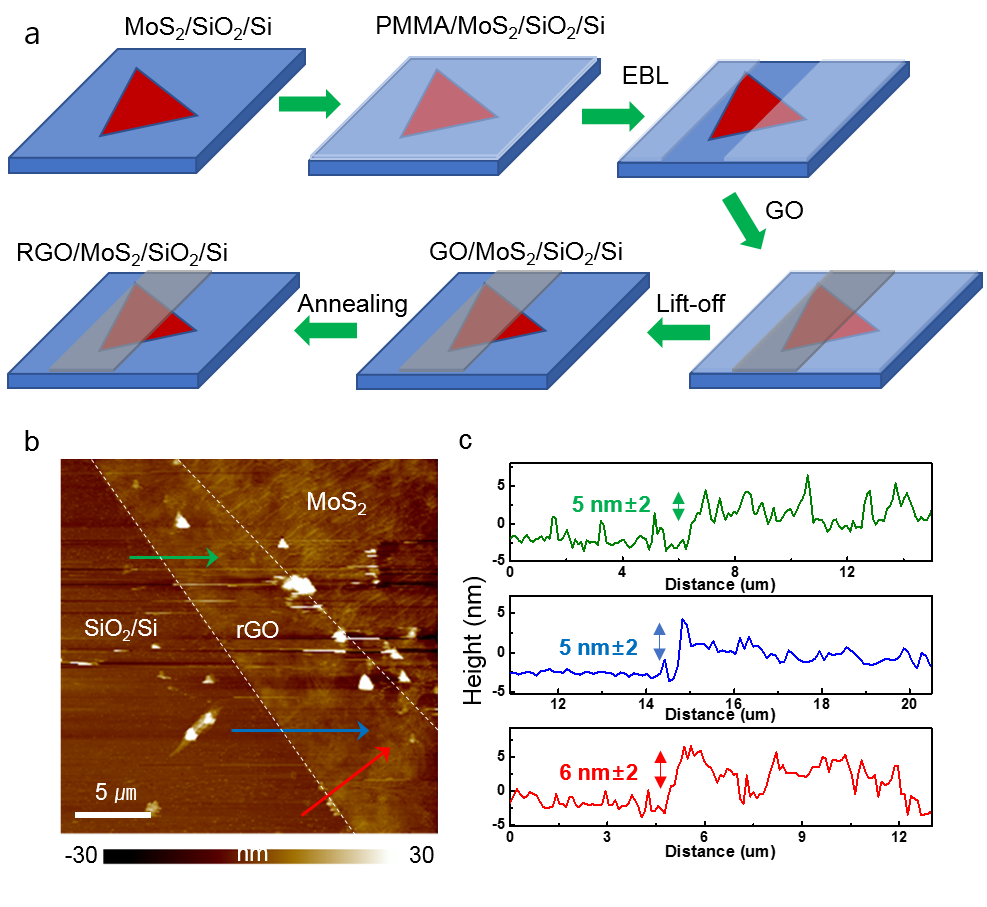


**Figure S4**. (a) Schematic of patterned rGO on MoS_2_ fabrication process. (b) AFM height image and (c) height profile of patterned rGO on MoS_2_ structure.

To confirm the thickness of coated rGO, we fabricated patterned rGO on MoS_2_ by e-beam lithography. Firstly, the CVD grown monolayer MoS_2_ transferred on SiO_2_/Si substrate by using the wet transfer. PMMA is spin coated on the MoS_2_ on substrate. E-beam lithography is then followed to produce a patterned mask and GO is spin coated on the sample. After the lift-off process, sample is annealed at 350 °C for 3 h.

Figure S5: Raman spectrum of the GO before and after thermal annealing.


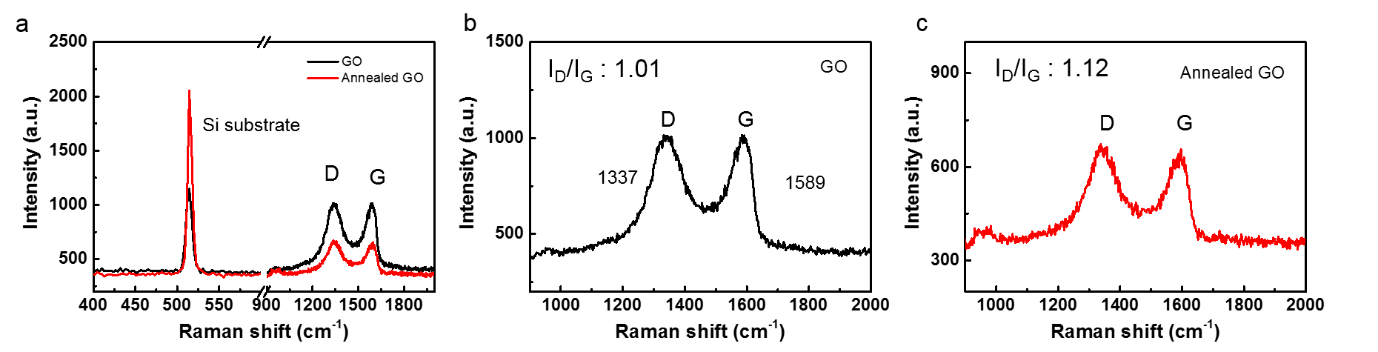


**Figure S5.** (a) Raman spectra of sample before and after annealing. (b) Raman spectrum of the GO before thermal annealing and (c) Raman spectrum of the GO after thermal annealing.

To investigate the reduction of GO, we carried out Raman spectroscopy measurement of the GO before and after thermal annealing treatment. As shown in Fig. S5b, GO and annealed GO exhibit two Raman peaks of the D band at 1337, and G band 1589 cm^−1^. We observed decrease of the intensity of spectrum and increased intensity ratio of the D to the G band (I_D_/I_G_) after thermal annealing. The I_D_/I_G_ ratio for GO was 1.01. After thermal annealing, the I_D_/I_G_ ratio of the sample become 1.12 which indicates the removal of most of the oxygen containing functional groups and the formation of large number of structural defects.^2^

Figure S6: Height profile of the stacked MoS_2_ with GO and rGO


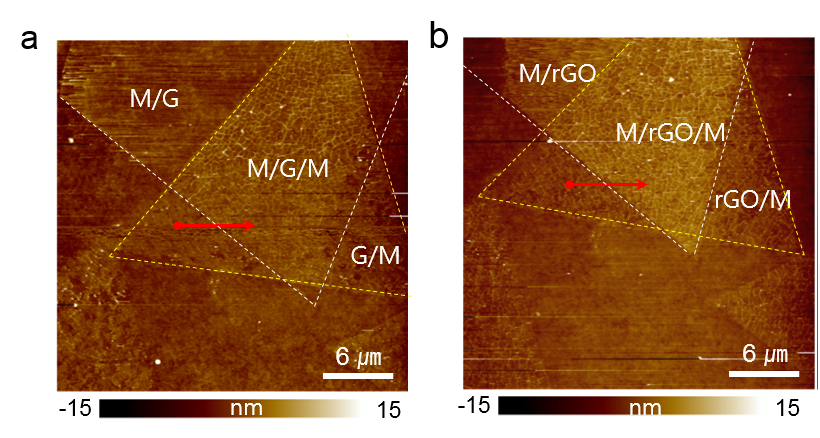


**Figure S6.** AFM image of (a) M/G/M and (b) M/rGO/M structure

Figure S6 (a) shows the AFM image of stacked bilayer MoS_2_ with GO (M/G/M), GO on monolayer MoS_2_ (G/M), and monolayer MoS_2_ on G (M/G) regions. Based on the AFM results, we observed wrinkles and some bubbles on the stacked MoS_2_ with GO. Figure S6 (b) shows the AFM image of stacked bilayer MoS_2_ with GO after thermal annealing. As shown in Figure S6 (b), we observed that wrinkles and bubble still existed after thermal annealing.

Figure S7: Height profile of the stacked MoS_2_ with rGO


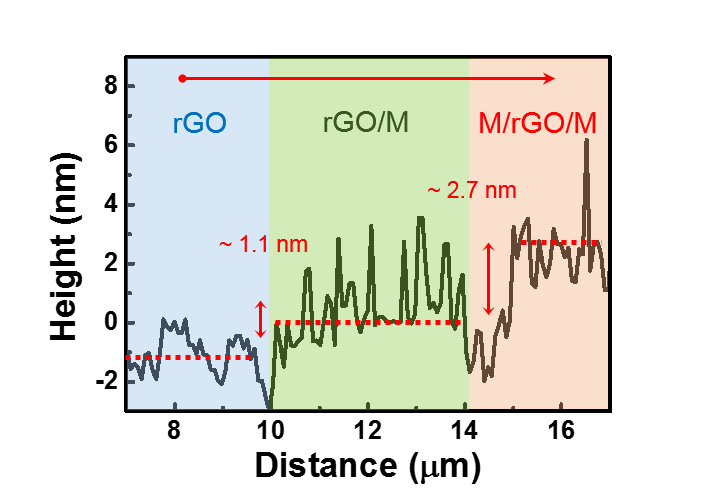


**Figure S7**. Height profile of the stacked MoS_2_ with rGO

Figure S8: PL intensity map and PL spectra stacked MoS_2_ with rGO.


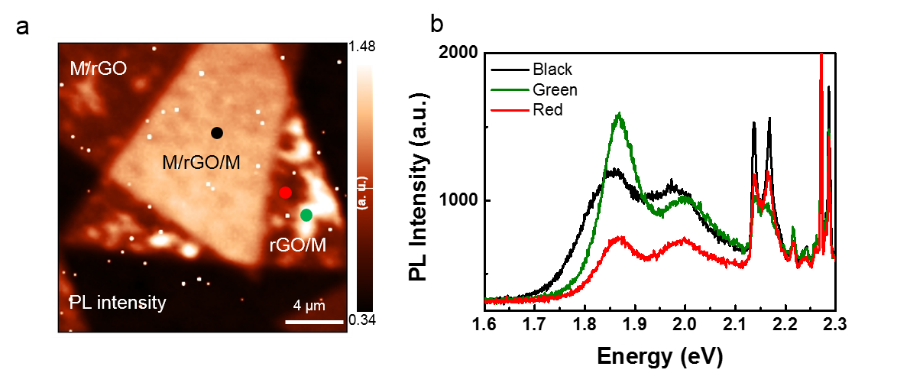


**Figure S8.** (a) PL intensity map image of stacked MoS_2_ with rGO. (b) PL spectra of the various position of the stacked MoS_2_ with rGO.

Figure S9: Raman spectra of the stacked MoS_2_ with GO or rGO.


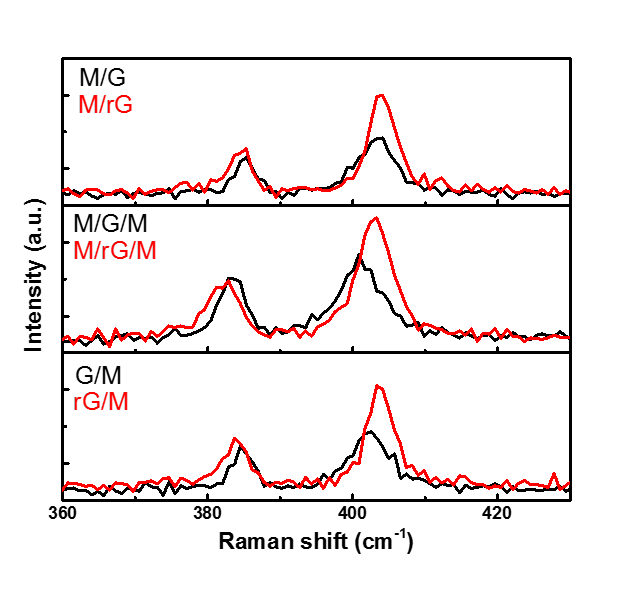


**Figure S9**. Raman spectra of the various position of stacked MoS_2_ with GO and rGO

**Reference**

1. Katzenmeyer, A. M., Aksyuk, V. & Centrone, A. *Anal. Chem.***85**, 1972-1979 (2013).

2. A. K. Das, M. Srivastav, R. K. Layek, M. E. Uddin, D. Jung, N. H. Kim and J. H. Lee, *J. Mater. Chem. A* **2**, 1332-1340 (2014)
